# IL-33 Promotes the Development of Colorectal Cancer Through Inducing Tumor-Infiltrating ST2L^+^ Regulatory T Cells in Mice

**DOI:** 10.1177/1533033818780091

**Published:** 2018-06-27

**Authors:** Yaxing Zhou, Yong Ji, Honggang Wang, Hai Zhang, Haihua Zhou

**Affiliations:** 1 Department of Hepatobiliary Surgery, the Fifth Affiliated Hospital of Medical School of Nantong University, Nantong, China; 2 Department of Cardiothoracic Surgery, Wuxi People’s Hospital Affiliated to Nanjing Medical University, Nanjing, China; 3 Department of Hepatobiliary Surgery, Affiliated Hospital of Jiangsu University, Zhenjiang, China

**Keywords:** Treg, IL-33, ST2L, colorectal cancer

## Abstract

Colorectal cancer, one of the most commonly diagnosed and lethal cancers worldwide, is accompanied by the disorders of immune system. However, the underlying mechanism is still not fully understood. In this study, our goal was to determine whether interleukin 33 promotes tumorigenesis and progression of colorectal cancer through increased recruitment of tumor-infiltrating ST2^+^ regulatory T cells in CT26 tumor-bearing mice. We found that the mRNA or protein levels of interleukin 33, soluble ST2, and membrane ST2 were elevated in the serum of tumor-bearing mice when compared to WT mice. The mRNA levels of interleukin 33, soluble ST2, and membrane ST2 were also elevated in the tissue of tumor-bearing mice when compared to surrounding nontumor muscular tissues. In addition, the frequency of ST2L^+^ regulatory T cells was significantly increased in both tumor tissue and spleen of tumor-bearing mice. Higher protein levels of interleukin-4, -10, and -13 were also observed in the serum or the tumor homogenates of tumor-bearing mice. We found exogenously administered recombinant mouse interleukin 33 promoted tumor size and induced tumor-infiltrating ST2L^+^ regulatory T cells in tumor-bearing mice while neutralizing interleukin-33 or ST2L inhibited tumor size and decreased ST2L^+^ regulatory T cells. Furthermore, ST2L^+^ regulatory T cells from tumor tissue were also able to suppress CD4^+^CD25^−^T cell proliferation and interferon γ production. Altogether, our findings demonstrate the critical roles of interleukin 33 in promoting colorectal cancer development through inducing tumor-infiltrating ST2L^+^ regulatory T cells, and inhibition of interleukin-33/ST2L signaling maybe a potential target for the prevention of colorectal cancer.

## Introduction

Colorectal carcinoma (CRC) represents one of the major forms of cancer, which has been intensely studied for tumor–immune interactions to develop successful immunotherapies. Once solid tumors develop, tumor-associated antigens elicit a host-mediated immune response characterized by tumor-infiltrating T lymphocytes.^1^ Patients with CRC are able to induce an antigen-specific T-cell response without prior immunotherapy.^2^ Elevated numbers of intratumoral cytotoxic (CD8^+^) and memory T cells (CD45RO^+^) have been shown to correlate with an aggressive phenotype reflected by tumor microinvasive status.^3^ In particular, many studies indicate that a high density of tumor-infiltrating regulatory T (Treg) cells correlates with the outcome of CRC.^4,5^ However, the role of tumor-infiltrating Treg cells on the development of CRC is not fully understood.

Treg cells are a group of heterogeneous T cells that usually coexpress CD4, CD25, and transcription factor forkhead box protein 3 (Foxp3) and play critical roles in suppressing the development of an effective immune response especially against cancer or infectious agents.^6^ Increased numbers of blood and tumor Treg have also been found in human CRC.^7^ Systemic removal of Tregs using anti-CD25 antibody resulted in tumor rejection and improved vaccine-induced antitumor T-cell responses in mouse model of CRC.^8^ Recently, interleukin (IL)-33/ST2L signaling has been found to be critical for expansion of Treg cells.^9^ ST2L^−/−^Treg cells are significantly impaired in their ability to prevent colonic inflammation and cellular infiltration,^10^ indicating that ST2L^+^Treg cells have important roles in their capacity to restrain inflammation. Cui *et al* showed that the expression of IL-33/ST2L in adenomas and CRC tissues was increased both in tumor stromal cells and in adenomatous/cancerous cells.^11^ Liu *et al* clarified that higher expressions of IL-33 and ST2L in poorly differentiated human CRC cells and enhanced IL-33/ST2L signaling promoted human CRC metastasis.^12^ Zhang *et al* found that IL-33 induced the enhanced recruitment of CD11b^+^GR1^+^ and CD11b^+^F4/80^+^ myeloid cells to remodel the tumor microenvironment by increased expression of mobilizing cytokines and tumor angiogenesis by activating endothelial cells.^13^ However, the expression and the potential role of tumor-infiltrating ST2L^+^Treg cells in CRC are still unknown.

In this study, we explored the changes in the tumor-infiltrating ST2L^+^Treg cells and related cytokines to demonstrate ST2L^+^Treg functional imbalance in mouse model of CRC. And for the first time, we found that blocking of IL-33 or ST2L reduced the tumor size accompany by decreasing serum IL-10 level in CT26 tumor-bearing mice.

## Materials and Methods

### Animals, Cells, and Tumors

Seventy-five 6-week-old Balb/c female mice, weighing 20 to 22 g, purchased from SLAC Laboratory Animal Co Ltd (Shanghai, China) were used in this study. The mice were free from specified pathogens. Experiments were performed in the SPF Animal Laboratory.

Mouse colon adenocarcinoma cell line (CT26) was obtained from Shanghai Bogoo Biological Technology Co, Ltd. Cells were cultivated in RPMI-1640 culture medium containing 10% new born calf serum, penicillin G, and streptomycin at 37°C in an 5% CO_2_ incubator. CT26 cells at the logarithmic growth phase were used to mix up into a suspension (1 × 10^6^/200 μL) and then were injected subcutaneously at day 0 in the right flank of Balb/c mice. And tumor growth was monitored once a week using a caliper. Volume was calculated using the formula: length × width^2^ × π/6.

### Quantitative Reverse Transcription Polymerase Chain Reaction

RNA was extracted from serum or tissue samples with RNeasy mini kit (Qiagen, Hilden, Germany). A total of 1 μg RNA was used for first-strand complementary DNA synthesis using SuperScript III reverse transcriptase (Invitrogen-Life Technologies, Carlsbad, California) and oligo(dT) primers. Polymerase chain reaction (PCR) was performed on the 7900HT fast real-time PCR system (Applied Biosystems-Life Technologies, Carlsbad, California). Data were normalized to endogenous housekeeping gene *GAPDH*, and relative changes were calculated using the ΔΔCt method with the control as the comparator. 5′-CACCCCTCAAATGAATCAGG-3′ and 5′-GGAGCTCCACAGAGTGTTCC-3′ for IL-33; 5′-ACGCTCGACTTATCCTGTGG-3′ and 5′-CAGGTCAATTGTTGGACACG-3′ for ST2; GTGATAGTCTTAAAAGTGTTCTGG-3′ and 5′-TCAAAAGTGTTTCAGGTCTAAGCA-3′ for ST2L; 5′-GGCAACCTGCCTAACATGCTT-3′ and 5′-CAAGTTGTCCAGCTGATCCTTCAT-3′ for IL-10; 5′-GCAGTCCTGGCTCTTGCTTG-3′ and 5′-TGCTTTGTGTAGCTGAGCAG-3′ for IL-13; 5′-ACGGAGATGGATGTGCCAAAC-3′, and 5′-AGCACCTTGGAAGCCCTACAGA′ for IL-4; and 5′-ACCACAGTCCATGCCATCAC-3′ and 5′-TCCACCACCCTGTTGCTGTA-3′ for the internal control GAPDH.

### Enzyme-Linked Immunosorbent Assay

Blood samples of mice were centrifuged for 30 minutes to separate serum (12 000 r/min). Levels of IL-4 (eBioscience, San Diego, California), IL-10 (eBioscience), interferon (IFN)-γ (eBioscience), IL-13 (eBioscience), IL-33 (Invitrogen), membrane-bound isoforms of ST2 (ST2L; Santa Cruz, California, USA), and soluble ST2 (sST2; Invitrogen) in serum were quantified with enzyme-linked immunosorbent assay (ELISA). In addition, the supernatant of tissue homogenate for the normal muscle or tumor tissue was also applied to detect IL-33 and sST2 by ELISA.

### Purified IL-33 or Neutralizing Anti-Mouse IL-33 or ST2L Antibody Administration

Interleukin 33 (5 μg per mouse; Promocell, Heidelberg, Germany) and phosphate-buffered saline (PBS) were administered intravenously (IV) into tumor-bearing mice 1 week following CT26 cells injection. The neutralizing rabbit antimouse IL-33 and ST2L antibody and their control immunoglobulin G (IgG) mAb were purchased from R&D systems (R&D, Minneapolis, MN). IL-33- or ST2L-neutralizing antibody (each 10 μg per mouse) was administered IV 1 week following CT26 cells injection (IV). All of the mice were performed once a week for 3 consecutive injections.

### Lymphocyte and Tumor Cell Collection for Flow Cytometry

Mice were anesthetized and killed to extract spleen cells. Briefly, single-cell suspensions of splenocytes were prepared by mincing the mouse spleen in PBS containing 1% fasting blood sugar (Gibco, Grand Island, New York) and 1% EDTA. Red blood cells were then lysed using red blood cell lysis buffer.

Fresh tumor tissues were washed 3 times in RPMI-1640 before being cut into small pieces and then digested in calf serum-free medium with 0.05% collagenase IV, 100 mg/L DNase I (Sigma, St Louis, Missouri), and 10 mg/mL DNase I (Roche, Basel, Switzerland). Tissue pieces were oscillated for 50 minutes in digestion buffer and then resuspended in RPMI-1640. After that, the suspended cells were added into mouse lymphocyte separation medium (Sigma) for further density gradient centrifugation, then the lymphocyte layer was collected for the following staining.

Single-cell suspensions of 1×10^6^ were surface stained with CD4-FITC, ST2L-APC, and CD25-PE-cyanine 7 (all from eBioscience). Then, cells were fixed and permeabilized with Cytofix/Cytoperm buffer (BD Pharmingen, San Diego, California). Finally, cells were blocked with Fc receptor (eBioscience) and then stained with phycoerythrin (PE)-conjugated anti-Foxp3 antibodies.

### Isolation of CD4^+^CD25^+^ST2L^+^ and CD4^+^CD25^−^ T Cells

CD4^+^CD25^+^ST2L^+^ T cells from tumor tissues were purified by flow sorting, while CD4^+^CD25^−^ T cells were purified from freshly isolated splenocytes by immunomagnetic sorting using mouse CD4^+^CD25^+^ Treg cell isolation kit (Miltenyi Biotec, Auburn, CA) and following the manufacturer’s instructions. The purity of the isolated cells was checked by surface staining with anti-CD4 and anti-CD25 mAb.

### ^3^H Thymidine Incorporation Assay

*In vitro* suppression assays were performed in 96-well round-bottom plates (Nalge Nunc, Rochester, New York). The responder CD4^+^CD25^−^ T cells were stimulated using anti-CD3/CD28 beads and incubated alone or with increasing numbers of freshly isolated autologous CD4^+^CD25^+^ST2L^+^ T cells. The proliferation of the responder T cells was evaluated 72 hours after the incubation of T suppressor cells with [^3^H]thymidine (Amersham Biosciences, Piscataway, New Jersey). [^3^H]thymidine was then added at 1 mCi per well for an additional 18 hours. In some experiments, supernatants were collected on day 2 for detecting cytokine profiling.

### Statistical Analysis

All analyses were carried out using SPSS 21.0 software. Data were shown as mean (SD). Comparisons among 4 groups were performed using 1-way analysis of variance, and Student-Newman-Keuls test was used for comparison between the 2 groups. The significant difference between the 2 groups was identified using a Student *t* test. Correlation analysis was made using 2-tailed Pearson correlation coefficient. *P* values <.05 were considered significant. Significant differences were as follows: *P < .05; **P < .01; ***P < .001.

## Results

### IL-33 and ST2L Is Induced in CT26 Tumor-Bearing Mice

**Figure 1.**
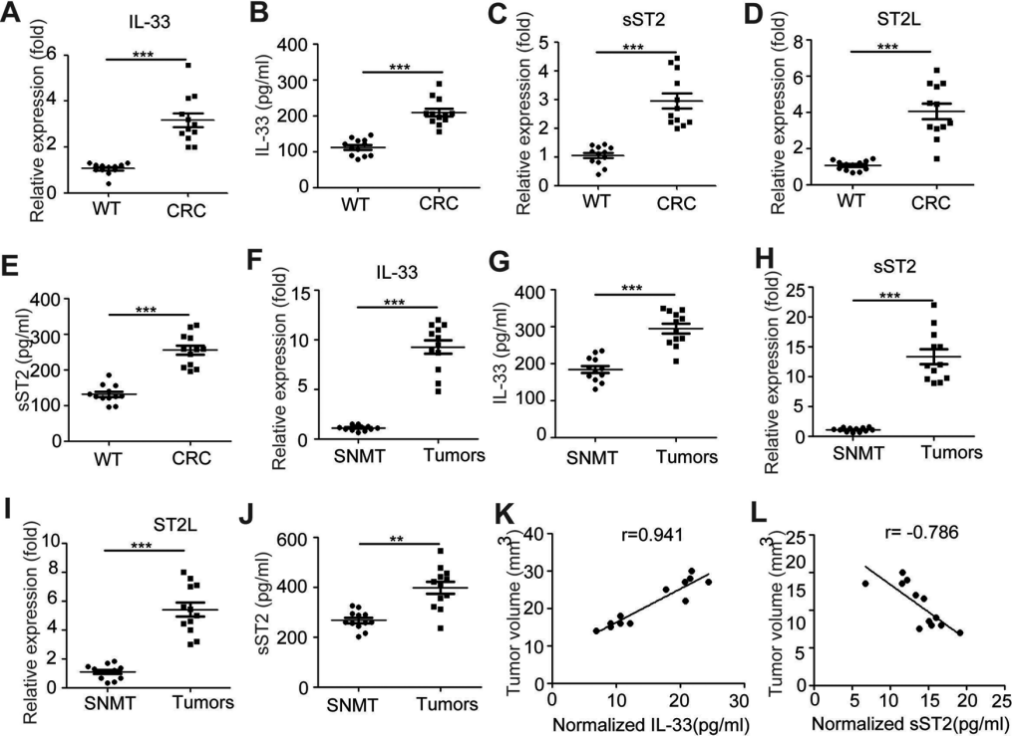
Increased expression of IL-33 and ST2L in the serum or tissue of CT26 tumor-bearing mice the mRNA levels of IL-33 (A), soluble ST2 (C), and ST2L (D) in the serum of WT and tumor-bearing mice were evaluated by qRT-PCR. The protein levels of IL-33 (B) and soluble ST2 (E) in the serum of WT and tumor-bearing mice were evaluated by ELISA. The mRNA levels of IL-33 (F), soluble ST2 (H), and ST2L (I) in the tumors or surrounding nontumor muscular tissues (SNMT) of tumor-bearing mice were evaluated by qRT-PCR; the protein levels of IL-33 (G) and soluble ST2 (J) in the tumor or SNMT homogenates of tumor-bearing mice were evaluated by ELISA. Data are from two independent experiments (mean [SD]), in which each experimental group contained 6 mice. ***P < .001 as compared to the control group (Student *t* test). **P < .01 as compared to the control group (Student *t* test). Pearson correlation analysis showed a correlation between the expression of normalized IL-33 or soluble ST2 (the protein levels/tumor volume) in the tumors and tumor volume (*P* < .05) (K, L). IL indicates interleukin; qRT-PCR, quantitative reverse transcription polymerase chain reaction; ELISA, enzyme-linked immunosorbent assay; SD, standard deviation.

To investigate the potential role of IL-33/ST2L pathway in the tumor development in a mouse model of CRC induced by CT26, we detected the messenger RNA (mRNA) and protein levels of IL-33 and sST2 or ST2L in the serum or tissue of tumor-bearing mice. As shown in Figure 1A and B, the serum mRNA and protein levels of IL-33 were elevated in the CRC mice when compared with WT mice (*P* < .05; *P* < .05, respectively); the mRNA and protein levels of sST2 or ST2L in the serum were elevated in CT26 tumor-bearing mice when compared to WT mice (*P* < .05; *P* < .05, respectively; Figure 1C-E). In addition, the mRNA levels of IL-33, sST2, and ST2L from tumors were significantly greater than from surrounding nontumor muscular tissues (Figure 1F-I); the protein levels of IL-33 and sST2 from tumor homogenates were also significantly increased than the surrounding nontumor muscular tissue homogenates (Figure 1G and J). Furthermore, the normalized levels of IL-33 in the tumor tissue homogenates were positively associated with the tumor size of tumor-bearing mice (*r* = 0.941, *P* < .05), while the normalized levels of sST2 were negatively associated with the tumor size (*r* = −0.786, *P* < .05; Figure 1I and J). Altogether, these data indicated that the IL-33/ST2L pathway is involved in CRC development in tumor-bearing mouse model.

### Enhanced Expression of ST2L^+^Treg Cells in CT26 Tumor-Bearing Mice

**Figure 2.**
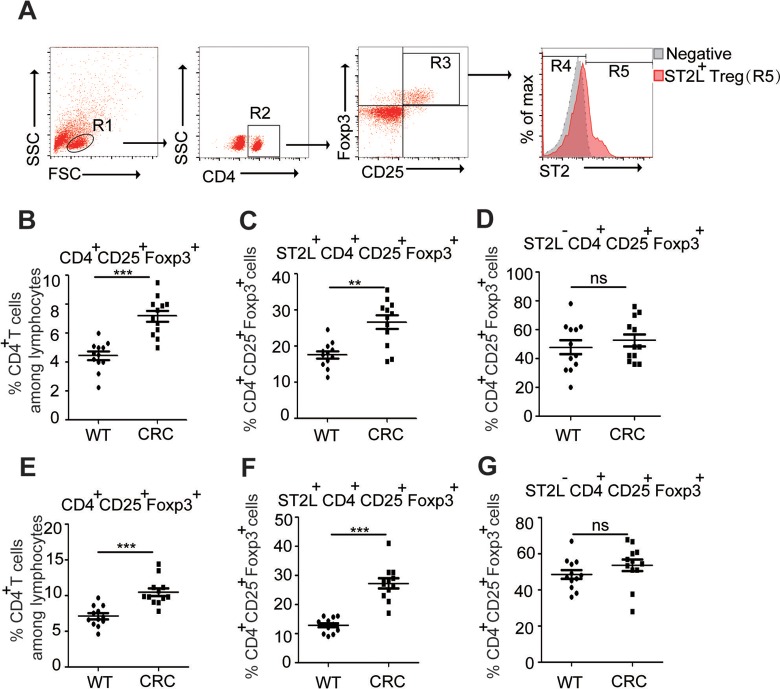
Enhanced expression of ST2L^+^Treg cells in CT26 tumor-bearing mice single-cell suspensions of spleens from normal or tumor-bearing mice were prepared. Gating schemes for analysis of the percentages of Treg (R3, gated from R1 and R2) and ST2L^+^Treg cells (R4, gated from R1, R2, and R3) (A). Cells in the spleen (B-D) or tumor tissues (E-G) were stained with CD4-FITC, ST2L-APC, and CD25-PE-cyanine 7 and then intracellularly stained with PE-conjugated antibodies against Foxp3 for FACS analysis of CD4^+^CD25^+^Foxp3^+^ (Treg) (B, E), ST2L^+^CD4^+^CD25^+^Foxp3^+^ (ST2L^+^Treg) (C, F), or ST2L^−^CD4^+^CD25^+^Foxp3^+^ (ST2L^−^Treg) (D, G) cells. Data are expressed as the mean (SD) of 12 mice for each group from 2 experiments (Student *t* test). Treg indicates regulatory T; PE, phycoerythrin; FACS, fluorescence-activated cell sorting; SD, standard deviation.

Treg cells, which play critical roles in suppressing the development of an effective immune response against CRC,^7^ were significantly increased in the spleen or the tumor tissue of tumor-bearing mice when compared to normal mice (*P* < .05; Figure 2, B and E). Given the critical roles of ST2L in the expansion of Treg cells,^9^ we tested the hypothesis that the expression of ST2L serves an important role in the development of CRC. Results showed that the percentage of ST2L^+^Treg cells was significantly increased in the spleen or tumor tissue of tumor-bearing mice when compared to normal mice (*P* < .05; Figure 2C and F); however, the frequency of ST2L^−^Treg cells did not change (Figure 2D and G). Altogether, these results suggested that ST2L^+^Treg cell maybe the main cell population that involved in tumor development.

### Enhanced Expression of Anti-inflammatory Factors in CT26 Tumor-Bearing Mice

**Figure 3.**
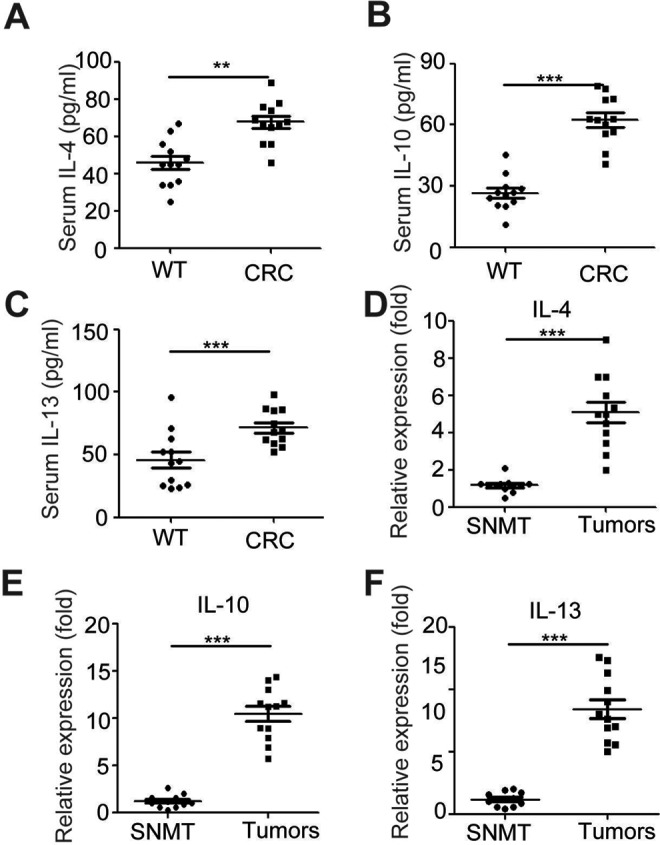
Enhanced expression of IL-10 and IL-13 in CT26 tumor-bearing mice the serum was purified from normal and tumor-bearing mice. The levels of Th2-related cytokine (IL-4, IL-10, and IL-13) (A-C) in serum were evaluated by ELISA. D-F, The mRNA levels of Th2-related cytokine (IL-4, IL-10, and IL-13) in the homogenates from tumors of tumor-bearing mice and surrounding nontumor muscular tissues (SNMT) were evaluated by ELISA. Data are expressed as the mean (SD) of 12 mice for each group from 2 experiments. **P < .01 as compared to the control group (Student *t* test). IL indicates interleukin; ELISA, enzyme-linked immunosorbent assay; SD, standard deviation.

As IL-33/ST2L signaling is linked to promote Th2 effector functions,^14^ we further evaluated Th2-related cytokines in CT26 tumor-bearing mice. Results showed that the protein levels of IL-4, IL-10, and IL-13 were significantly increased in the serum of tumor-bearing mice, respectively (*P*s < .05; Figure 3A-C). In addition, the mRNA levels of IL-4, IL-10, and IL-13 were significantly higher in the tumor tissues when compared to nontumor muscular tissues (*P*s < .05; Figure 3D-F).

### IL-33 Promote the Tumor-Infiltrating ST2L^+^Treg Cells in CT26 Tumor-Bearing Mice

**Figure 4.**
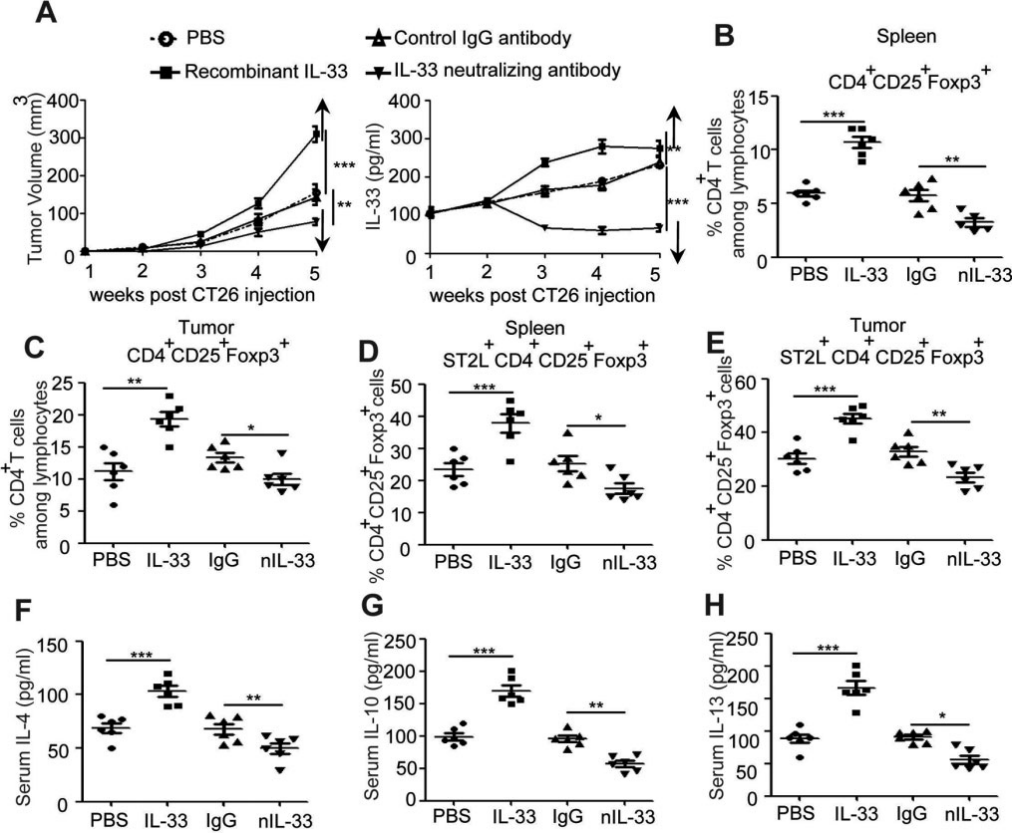
IL-33 enhanced tumor size and antiinflammatory factor purified IL-33 and IL-33-neutralizing antibody were administered into tumor-bearing mice, and dynamic serum IL-33 levels or tumor volume were observed (1, 2, 3, 4, and 5 weeks after IL-33 injection via IV injection) (A). Single-cell suspensions of spleens or tumor tissues from control PBS group, IL-33 treatment group, control IgG-treated group, and IL-33-neutralizing antibody-treated group were prepared. Cells were stained with CD4-FITC, ST2L-APC, and CD25-PE-cyanine 7 and then intracellularly stained with PE-conjugated antibodies against Foxp3 for FACS analysis of CD4^+^CD25^+^Foxp3^+^ (Treg) (B, C) and ST2L^+^CD4^+^ CD25^+^Foxp3^+^ (ST2L^+^Treg) (D, E). The serum from above mice was purified for detecting the serum levels of Th2-related cytokine (IL-4, IL-10, and IL-13) by ELISA (F-H). ***P < .001, **P < .01, ANOVA/SNK. IL indicates interleukin; IV, intravenous; PBS, phosphate buffer serum; IgG, immunoglobulin G; Treg, regulatory T; PE, phycoerythrin; ELISA, enzyme-linked immunosorbent assay; FACS, fluorescence-activated cell sorting; ANOVA, analysis of variance; SNK, Student-Newman-Keuls.

**Figure 5.**
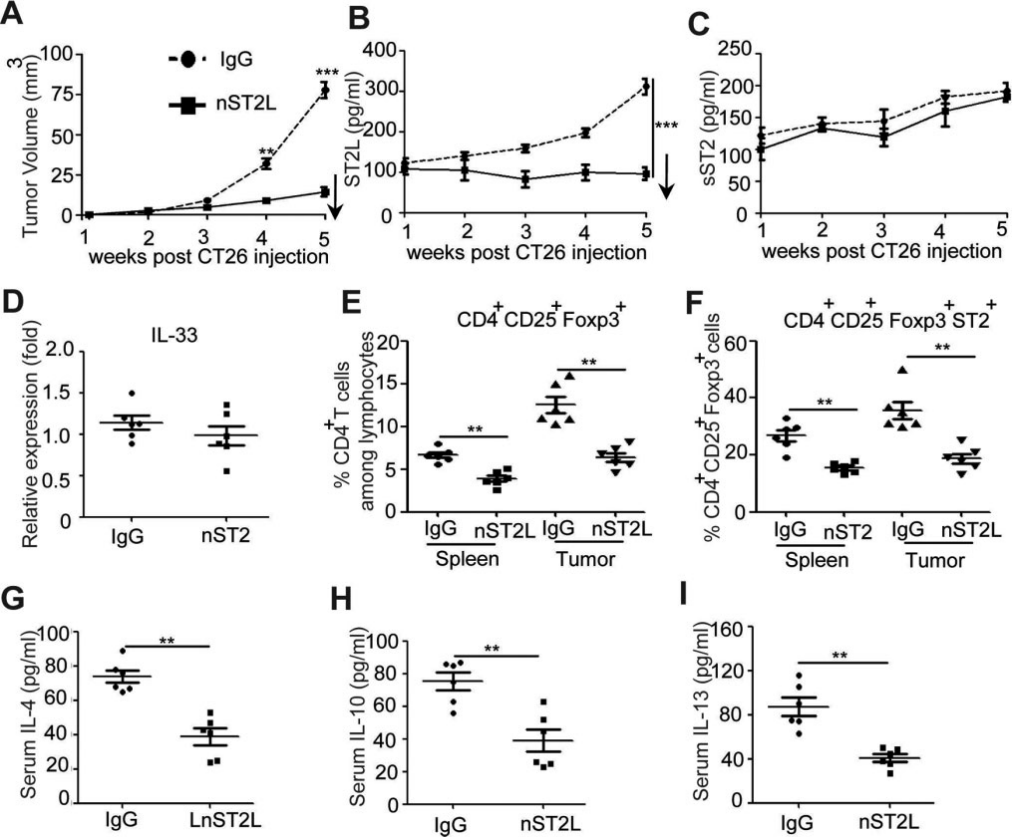
Neutralization of ST2L reduced tumor size and antiinflammatory factor rabbit anti-mouse ST2L antibody or controls were administered to tumor-bearing mice. The dynamic tumor volume was observed (1, 2, 3, 4, and 5 weeks after ST2L neutralization) (A). The changes of sST2, ST2L, and IL-33 in the serum were detected by ELISA or qRT-PCR (B-D). Single-cell suspensions of the spleens or tumor tissues from control IgG group and ST2 neutralization group were prepared. Cells were stained with CD4-FITC, ST2L-APC, and CD25-PE-cyanine 7 and then intracellularly stained with PE-conjugated antibodies against Foxp3 for FACS analysis of CD4^+^CD25^+^Foxp3^+^ (Treg) (E) and ST2L^+^CD4^+^ CD25^+^Foxp3^+^ (ST2L^+^Treg) (F). The serum from above mice was purified and detected the serum levels of Th2-related cytokine (IL-4, IL-10, and IL-13) by ELISA (F-G). (A-C, F-H) ***P < .001, Student *t* test; (G-I) **P < .01, ANOVA/SNK. IL indicates interleukin; sST2, soluble ST2; ELISA, enzyme-linked immunosorbent assay; qRT-PCR, quantitative reverse transcription polymerase chain reaction; IgG, immunoglobulin G; PE, phycoerythrin; FACS, fluorescence-activated cell sorting; Treg, regulatory T; ANOVA, analysis of variance; SNK, Student-Newman-Keuls.

To evaluate the effects of IL-33 on CRC development, purified recombinant mouse IL-33 was administrated into tumor-bearing mice. Results showed that administration of IL-33 sustained elevated levels of IL-33 in the serum of tumor-bearing mice (Figure 4A), significantly increased tumor size (Figure 4A), and induced the percentage of Tregs (Figure 4B and C) as well as ST2L^+^Treg cells (Figure 4D and E) in both spleen and tumor tissue of tumor-bearing mice. The antiinflammatory factors, including IL-4, IL-10, and IL-13, in IL-33-treated tumor-bearing mice were also significantly increased (Figures 4F-5H). In addition, IL-33-neutralizing antibody was injected into tumor-bearing mice to further clarify its roles on CRC development, and results showed that neutralization of IL-33 significantly decreased serum levels of IL-33 and tumor size (Figure 4A), dampened Treg and ST2L^+^Treg cells in tumor-bearing mice (Figure 4B-E) as well as decreased antiinflammatory factors (Figures 4F-5H). Altogether, these results suggested that IL-33 induced the recruitment of tumor-infiltrating ST2L^+^Treg cells that contributed to the development of CRC.

### Neutralization of ST2L Reduce the Tumor Formation of CRC

To examine the effects of ST2L on the development of CRC, ST2L-neutralizing antibody was administrated into tumor-bearing mice. Compared to the control IgG group, neutralization of ST2L significantly decreased the tumor size in tumor-bearing mice (*P* < .05; Figure 5A), accompanied by inhibition of the serum ST2L, but not sST2 and IL-33, suggesting neutralization of ST2L may not regulate sST2 and IL-33 expression (Figure 5B-D). In addition, neutralization of ST2L significantly decreased Treg cells and ST2L^+^Treg both in spleen and in tumor tissue of tumor-bearing mice (Figure 5E and F) and inhibited the protein levels of IL-4, IL-10, and IL-13 (Figure 5G-I). Together, these results suggested that ST2L signaling plays a critical role in promoting the development of CRC.

### ST2L^+^Tregs Isolated From the Tumor Tissue Suppress Autologous CD4^+^CD25^−^ T-Cell Proliferation and IFN-γ Production *In Vitro*

**Figure 6.**
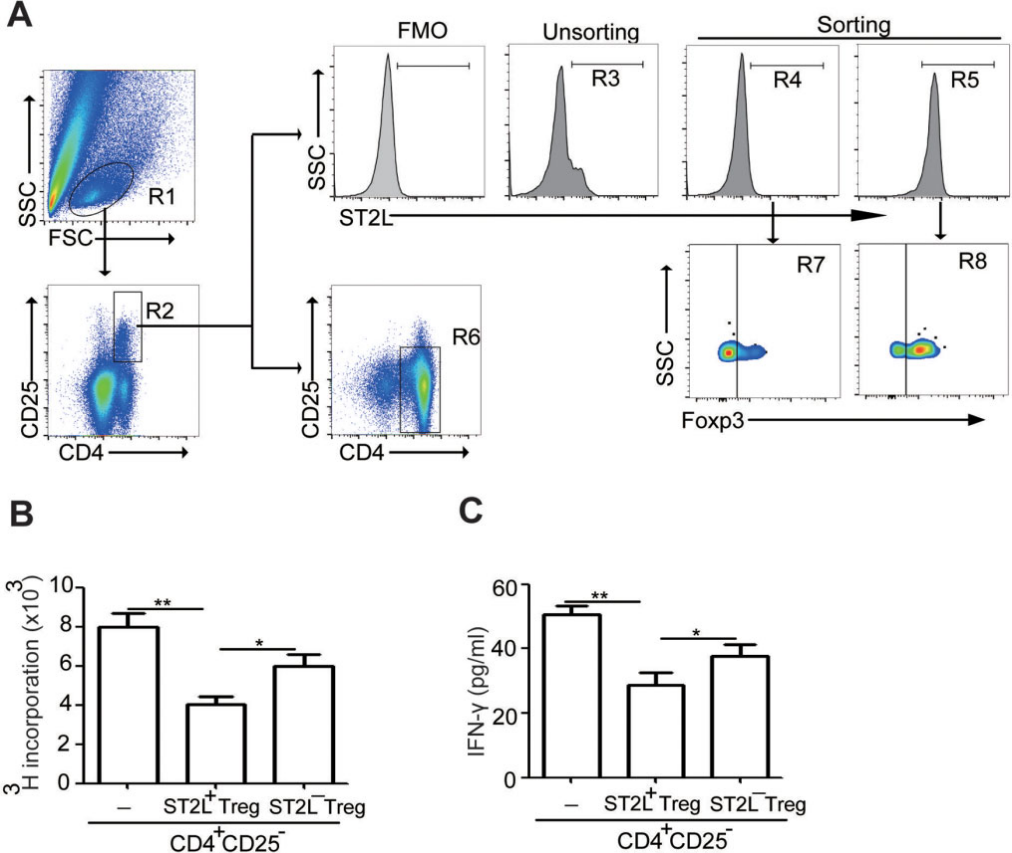
ST2L^+^Tregs suppress CD4^+^CD25^−^ T-cell proliferation and IFN-γ production single-cell suspensions of tumor tissue from tumor-bearing mice were prepared. Gating schemes for analysis of the percentages of ST2L^+^CD4^+^CD25^+^ cells (R5, gated from R1 and R2) or ST2L^−^CD4^+^CD25^+^ cells (R4, gated from R1 and R2) after purification by flow sorting (A) or spleen CD4^+^CD25^−^ cells (R6, gated from R1) after purification by immunomagnetic sorting. Cells were stained with CD4-FITC, ST2L-APC, and CD25-PE-cyanine 7 and then intracellularly stained with PE-conjugated antibodies against Foxp3 for FACS analysis. Freshly sorted tumoral ST2L^+^CD4^+^CD25^+^ cells or ST2L^−^CD4^+^CD25^+^ cells were used in suppression assays, and the proliferation of spleen CD4^+^CD25^−^ cells (B) or the IFN-γ secretion (C) was assessed with and without ST2L^+^CD4^+^CD25^+^ or ST2L^−^CD4^+^CD25^+^ cells at 1:2 ratio. The experiments were Performed twice, with similar results (**P < .01, Student *t* test). Treg indicates regulatory T; IFN-γ, interferon-γ; PE, phycoerythrin; FACS, fluorescence-activated cell sorting.

To further determine the suppressive capacity of ST2L^+^Treg or ST2L^−^Treg cells, we sorted ST2L^+^CD4^+^CD25^+^ (R5 in Figure 6A, ∼98% purity) and ST2L^−^CD4^+^CD25^+^ (R4 in Figure 6A, ∼99% purity) T cells from CRC tissue (Figure 6A-C). Results showed that ∼80% of ST2L^+^CD4^+^CD25^+^ cells (R8 in Figure 6A) and 20% of ST2L^−^CD4^+^CD25^+^ cells (R7 in Figure 6A) expressed Foxp3, a specific molecular marker for Tregs. In addition, the suppressive roles of ST2L^+^CD4^+^CD25^+^ T cells on CD4^+^CD25^−^ T (R6 in Figure 6A, ∼90% purity) cell proliferation were much more than ST2L^−^CD4^+^CD25^+^ T cells (Figure 6B). Furthermore, ST2L^+^CD4^+^CD25^+^ T cells also efficiently suppressed production of the proinflammatory cytokines IFN-γ by CD4^+^CD25^−^ T cells from tumor tissue, while ST2L^−^CD4^+^CD25^+^ T cells had milder suppressive function (Figure 6C). Altogether, these results suggested that ST2L**^+^**Tregs contributed to the inhibitory function in the tumor microenvironment.

## Discussion

Although it has been well recognized that Treg cells play an important role in the progress of CRC, the role of IL-33/ST2L signaling in Treg cells on CRC development remains not fully understood. In this study, we found that ST2L^+^Treg cells play critical roles in the development of CRC, and blockade of IL-33 or ST2L could be a potential target for the prevention of CRC.

CD4^+^CD25^+^Foxp3^+^Treg cells, characterized by increased expression of the surface markers CTLA-4, CD45RO, and GITR,^15-18^ are abundant in the blood and populate the tumor mass and draining lymph nodes of patients with many different cancers.^19,20^ It is now appreciated that Tregs can inhibit tumor-specific CD8^+^ and CD4^+^ T-cell effector functions through cell–cell contact and/or the production of soluble factors such as IL-10 or transforming growth factor β.^21,22^ The increased frequency of Treg cells was observed in the peripheral blood of patients with various types of cancer, including CRC.^4,5,23^ Depletion in Tregs promotes the generation of antitumor immune responses and tumor rejection in humans and murine model.^24-26^ Recently, it was shown that ST2L is also expressed on a subset of Tregs,^10^ and ST2L^+^Treg cells had significant roles in their capacity to restrain inflammation.^9,10^ However, the potential roles of ST2L^+^ Treg cells in the development of CRC are still unknown.

IL-33 is constitutively expressed in certain epithelial, endothelial, and fibroblastic cells, wherein it binds to chromatin and influences gene expression.^27^ Recent studies in humans have described high levels of IL-33 in CRC tissues,^11,12^ suggesting a potential role for this specific cytokine in the pathogenesis of CRC. IL-33 regulates innate and acquired immunity through binding to ST2L molecule of the IL-33 R complex expressed on murine and human Th2 cells, natural killer cells, mast cells, dendritic cells (DCs), and myeloid cells.^28^ To assess whether IL-33/ST2L signaling affects the development of CRC, we detected the expression of IL-33, sST2, and ST2L in the serum of CT26 tumor-bearing mice. Results showed that the expression of IL-33, sST2, and ST2L in the serum or tumors was elevated in tumor-bearing mice. These results are in agreement with the observations derived from Liu *et al*’s study,^12^ which showed higher expressions of IL-33 and ST2L in poorly differentiated human CRC cells. However, the levels of sST2 were negatively associated with tumor size, which were corresponded to O’Donnell *et al*’s study.^29^ In addition, we found that the frequency of ST2L^+^Treg cells and related cytokines (IL-4, IL-10, and IL-13) in the CRC mice were significantly increased but not ST2L^−^Treg cells, suggesting that ST2L^+^Treg cells maybe the main subpopulation that play important roles in the immunosuppression and promoting the development of CRC. Recent study showed that IL-33 promotes breast cancer growth and metastases by amplification of ST2L^+^Treg cell populations in a model of breast cancer,^30^ which supported our findings.

He *et al* investigated the role of epithelial expressed IL-33 during development of intestinal tumors and found that the expression of IL-33 within epithelial cells promoted tumor development by using Apc^Min/+^ or *V33* Apc^Min/+^ transgenic mice.^31^ Our study examined the effects of IL-33/ST2L signaling in regulating tumor-infiltrating ST2L^+^Treg cells on CRC development after administration of recombinant IL-33 or neutralizing antibody. We found that the higher expressed IL-33 in tumor-bearing mice promoted the tumor size, increased ST2L^+^Treg cells, and antiinflammatory cytokines production, while neutralization of IL-33 inhibited tumor size decreased ST2L^+^Treg cells and antiinflammatory cytokines production. However, whether IL-33 induced expression if ST2L in tumor-infiltrating Tregs or IL-33 induced the recruitment of ST2L^+^Tregs into the tumor bed where they contribute to CRC development need further study. Similarly, IL-33 can promote antiinflammatory cytokines production in cutaneous fibrosis and inflammatory bowel disease, respectively.^32-34^ Rank *et al* found that IL-33-activated DCs prime naive CD4^+^ T cells to produce Th2-type cytokines.^34^ We also used neutralizing mouse anti-ST2L antibody to study the role of IL-33 downstream on the development of CRC. Results showed that decreased tumor size in tumor-bearing mice was observed after neutralization of ST2L, which confirmed the significant role of IL-33/ST2L signaling in tumor growth. In addition, neutralization of ST2L decreased the expression of ST2L, but not IL-33 and sST2, suggesting IL-33 functions upstream of ST2L^+^Treg cells. We also found that blockade of ST2L signaling resulted in decreased ST2L^+^Treg cells and related antiinflammatory cytokines, suggesting that the central roles of IL-33/ST2L signaling in prompting CRC development involved in antiinflammatory response. Kropf *et al* found that administration of anti-ST2L antibodies induced resistance to *Leishmania major* infection in Balb/c mice, enhanced Th1 responses as indicated by increased IFN-γ production and decreased IL-4 and IL-5 synthesis,^35^ which supported our results in mouse model of CRC.

ST2L^+^Treg cells had been reported to express high levels of KLRG1, CD103, and OX40, suggesting that they were activated and had regulatory functions.^10^ Suppression experiments performed after cell sorting of ST2L^+^CD4^+^CD25^+^ T cell from tumor specimens showed that they could suppress splenic CD4^+^ effector T-cell (CD4^+^CD25^–^) proliferation and Th1 cytokine secretion; however, ST2L^−^CD4^+^CD25^+^ T cells had lower suppression function. ST2L^−^Treg cells had been reported to impair their suppression ability to restrain inflammation,^10^ which supported our results. Together, these data demonstrate for the first time that a new ST2L^+^CD4^+^CD25^+^ T-cell subset with suppressive functions is present in colorectal tumors of mouse model.

In conclusion, our study demonstrates that ST2L^+^Treg cells do play a functional role in the generation of Th2 cytokine responses and suppression of Th1 response during the development of CRC. Our data suggested that inhibition of IL33/ST2L signaling is a potential therapeutic target for the prevention of CRC.

## Abbreviations

CRC: colorectal carcinoma
ELISA: enzyme-linked immunosorbent assay
IFN-γ: interferon-γ
IgG: immunoglobulin G
IL: interleukin
IV: intravenous
PBS: phosphate buffer serum
PCR: polymerase chain reaction
PE: phycoerythrin
ST2L: membrane ST2
sST2: soluble ST2
Treg: regulatory T
